# Directed evolution of a genetically encoded immobilized lipase for the efficient production of biodiesel from waste cooking oil

**DOI:** 10.1186/s13068-019-1509-5

**Published:** 2019-06-28

**Authors:** Bradley S. Heater, Wai Shan Chan, Marianne M. Lee, Michael K. Chan

**Affiliations:** School of Life Sciences & Center of Novel Biomaterials, The Chinese University of Hong Kong, Hong Kong, SAR China

**Keywords:** Biodiesel, Waste cooking oil, Enzyme immobilization, Directed evolution, Cry3Aa crystals, Enzyme stability

## Abstract

**Background:**

We have recently developed a one-step, genetically encoded immobilization approach based on fusion of a target enzyme to the self-crystallizing protein Cry3Aa, followed by direct production and isolation of the fusion crystals from *Bacillus thuringiensis*. Using this approach, *Bacillus subtilis* lipase A was genetically fused to Cry3Aa to produce a Cry3Aa–lipA catalyst capable of the facile conversion of coconut oil into biodiesel over 10 reaction cycles. Here, we investigate the fusion of another lipase to Cry3Aa with the goal of producing a catalyst suitable for the conversion of waste cooking oil into biodiesel.

**Results:**

Genetic fusion of the *Proteus mirabilis* lipase (PML) to Cry3Aa allowed for the production of immobilized lipase crystals (Cry3Aa–PML) directly in bacterial cells. The fusion resulted in the loss of PML activity, however, and so taking advantage of its genetically encoded immobilization, directed evolution was performed on Cry3Aa–PML directly in its immobilized state in vivo. This novel strategy allowed for the selection of an immobilized PML mutant with 4.3-fold higher catalytic efficiency and improved stability. The resulting improved Cry3Aa–PML catalyst could be used to catalyze the conversion of waste cooking oil into biodiesel for at least 15 cycles with minimal loss in conversion efficiency.

**Conclusions:**

The genetically encoded nature of our Cry3Aa-fusion immobilization platform makes it possible to perform both directed evolution and screening of immobilized enzymes directly in vivo. This work is the first example of the use of directed evolution to optimize an enzyme in its immobilized state allowing for identification of a mutant that would unlikely have been identified from screening of its soluble form. We demonstrate that the resulting Cry3Aa–PML catalyst is suitable for the recyclable conversion of waste cooking oil into biodiesel.

**Electronic supplementary material:**

The online version of this article (10.1186/s13068-019-1509-5) contains supplementary material, which is available to authorized users.

## Background

Human combustion of fossil fuels has resulted in increased atmospheric CO_2_. This is widely believed to have contributed to global warming and climate change [1, 2]. One strategy to reduce net carbon emissions to the atmosphere is to use carbon-neutral fuels, such as biodiesel comprised of fatty acid methyl esters (FAMEs) generated from vegetable oils. FAME is attractive as a biofuel since it is renewable, clean burning, and compatible with diesel engines, both directly and as a blend with traditional petrodiesel [3, 4].

**Fig. 1:**
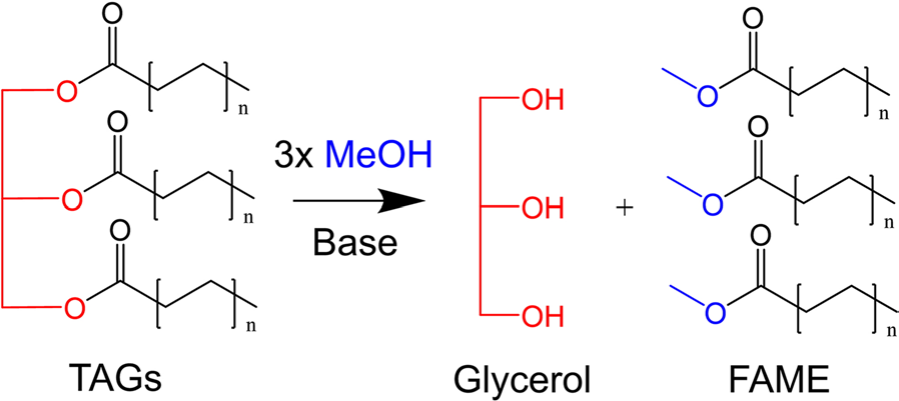
Schematic of FAME biodiesel synthesis via base-catalyzed transesterification of TAGs with MeOH

Currently, FAME biodiesel is most commonly produced using base-catalyzed transesterification of triacylglycerols (TAGs) with methanol (MeOH) (Fig. 1). Vegetable oil is a common feedstock for biodiesel production, though its relatively high price [5] and its negative impact on food security make it less appealing. With these concerns in mind, one attractive alternative is waste cooking oil (WCO). WCO is non-edible, cheap, and abundant. However, it contains a high content of free fatty acids (FFAs) that can form soap in the presence of a base catalyst. While an acid pre-treatment step can be applied to convert the FFAs into FAME prior to adding a base catalyst for TAG conversion, this increases the production costs and poses significant environmental concerns [6, 7].

An alternative conversion strategy to convert WCO into biodiesel is the use of lipases, since they can catalyze the conversion of both FFAs and TAGs into FAME biodiesel under mild conditions and temperatures (i.e., low energy input), and in the presence of water [8–10]. A key hurdle to the application of lipases for biodiesel production is their stability, reusability and production costs [11–13].

One common approach to address these issues is to immobilize the lipase, which permits for its easy removal and reuse during multiple cycles of biodiesel conversion. Most immobilization approaches used for biodiesel lipases involve multiple steps: (1) expression and purification of the lipase catalyst, (2) synthesis of the support, and (3) anchoring of the lipase on the support. One potential improvement is the use of genetically encoded immobilization approaches which allow for the direct production of immobilized enzymes in bacterial cells [14–16].

Lipase catalysts can also be improved by directed evolution to generate mutants with higher thermal and/or organic solvent stability [17–20], as well as enhanced MeOH tolerance specifically for biodiesel production [21–23]. One potential limitation of these previous studies is that the lipase is evolved in its soluble form, as opposed to its immobilized state used for catalysis. This could lead to discrepancies between enzyme function obtained from screening and enzyme function after immobilization. For instance, surface residue mutations could substantially increase an enzyme’s stability [24, 25], but these same mutations could alter how that enzyme behaves after immobilization. Therefore, an approach to directly evolve enzymes in their immobilized state could be beneficial.

Systems where evolution of the immobilized lipase could be potentially achieved include genetically encoded immobilization approaches. Here, since expression and immobilization occur simultaneously, any mutants produced can be directly screened in their immobilized state. Several fusion tags have been exploited to generate immobilized enzymes in vivo including self-aggregating peptides [26–28] and protein domains [16, 29, 30]. However, to the best of our knowledge, there has been no report of directed evolution studies performed on such systems.

Our contribution to these genetically encoded approaches involves the genetic fusion of enzymes to the self-crystallizing protein Cry3Aa [31–33], and their production as immobilized Cry3Aa-fusion crystals (CFCs) in *Bacillus thuringiensis* (*Bt*) [34, 35]. We showed that genetic fusion of Cry3Aa to lipase A from *Bacillus subtilis* (lipA) resulted in Cry3Aa–lipA crystals capable of catalyzing the conversion of coconut oil to biodiesel with high efficiency over 10 reactions cycles [34]. This work was the first example of using a genetically encoded immobilized lipase for biodiesel production with both high activity and recyclability. Unfortunately, this Cry3Aa–lipA catalyst was non-ideal for WCO since lipA prefers medium-chain (C_6_–C_12_) fatty acids as substrates [36]; while WCO is mostly comprised of long-chain (C_14_–C_22_) fatty acids [37]. We thus decided to explore the properties of Cry3Aa fused to other lipases with a natural substrate preference for long-chain fatty acids.

**Fig. 2:**
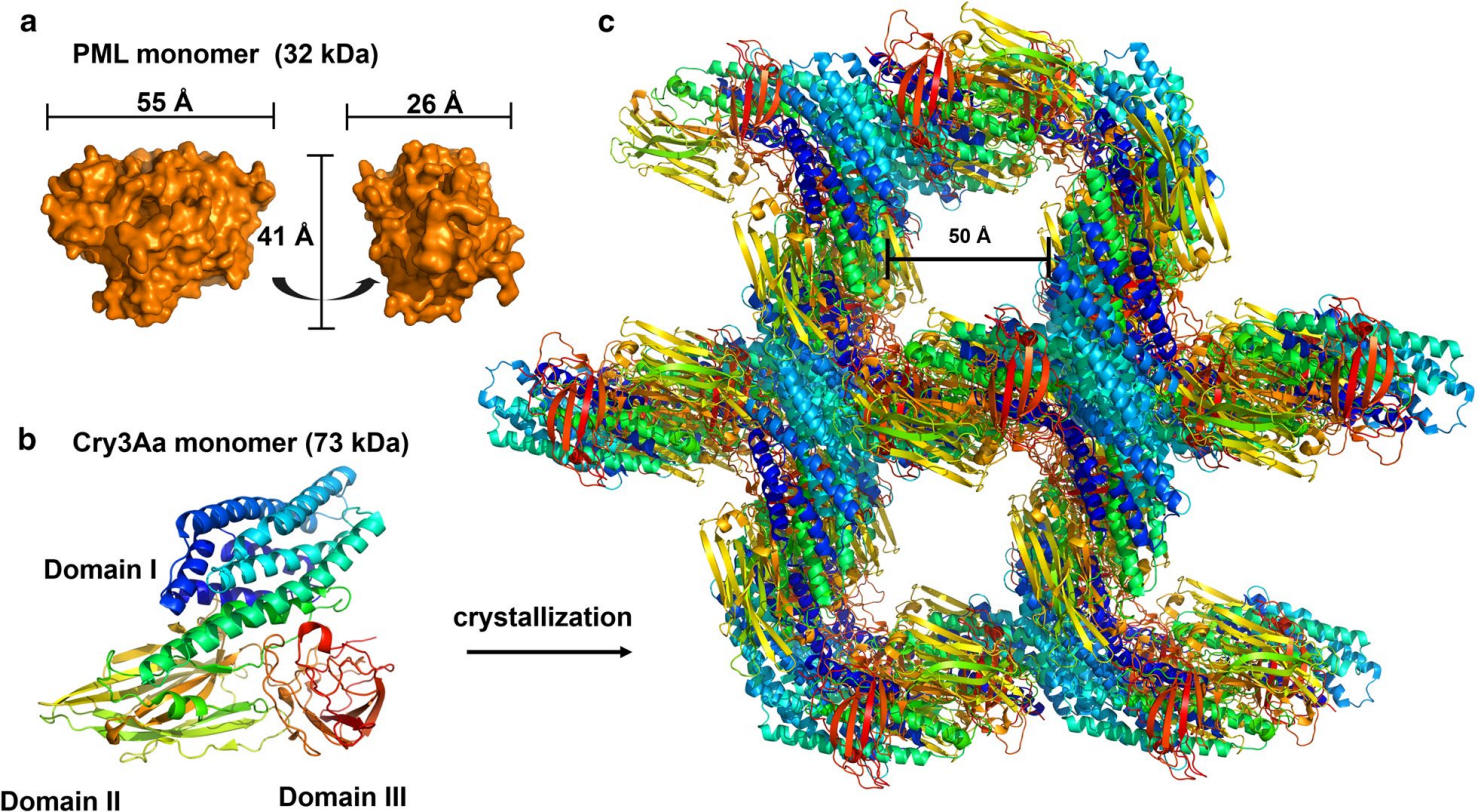
Schematic showing how PML could be accommodated in the Cry3Aa channel. **a** Dimensions of the PML fusion partner. **b** Structure of Cry3Aa [38]. Cry3Aa is an insecticidal protein used by *Bacillus thuringiensis* and contains three structural domains: a seven-helix bundle (Domain I), a three-sheet domain (Domain II), and a β sandwich (Domain III). **c** Cry3Aa self-assembles into protein crystals containing large solvent channels (50 Å by 50 Å)

This directed our attention to *Proteus mirabilis* lipase (PML) for fusion to Cry3Aa as a potential biodiesel catalyst for WCO. We surmised that PML would be suitable for Cry3Aa fusion, since it expresses well in *E. coli*, does not require a signal peptide for proper folding, and its size (55 Å × 41 Å × 26 Å) is small enough to be accommodated in the natural channels present in the Cry3Aa crystal (Fig. 2). Moreover, an improved mutant of PML, Dieselzyme 4 (DLZM4), with high MeOH tolerance had been identified by directed evolution of PML in its soluble state [21]. Thus, the use of PML was attractive since it would allow for comparison of the mutants produced from evolution in the immobilized versus soluble state. Herein, we report the properties of the resulting Cry3Aa–PML catalyst and its evolved mutant.

## Results and discussion

### Production and characterization of Cry3Aa-fusion crystals

**Fig. 3:**
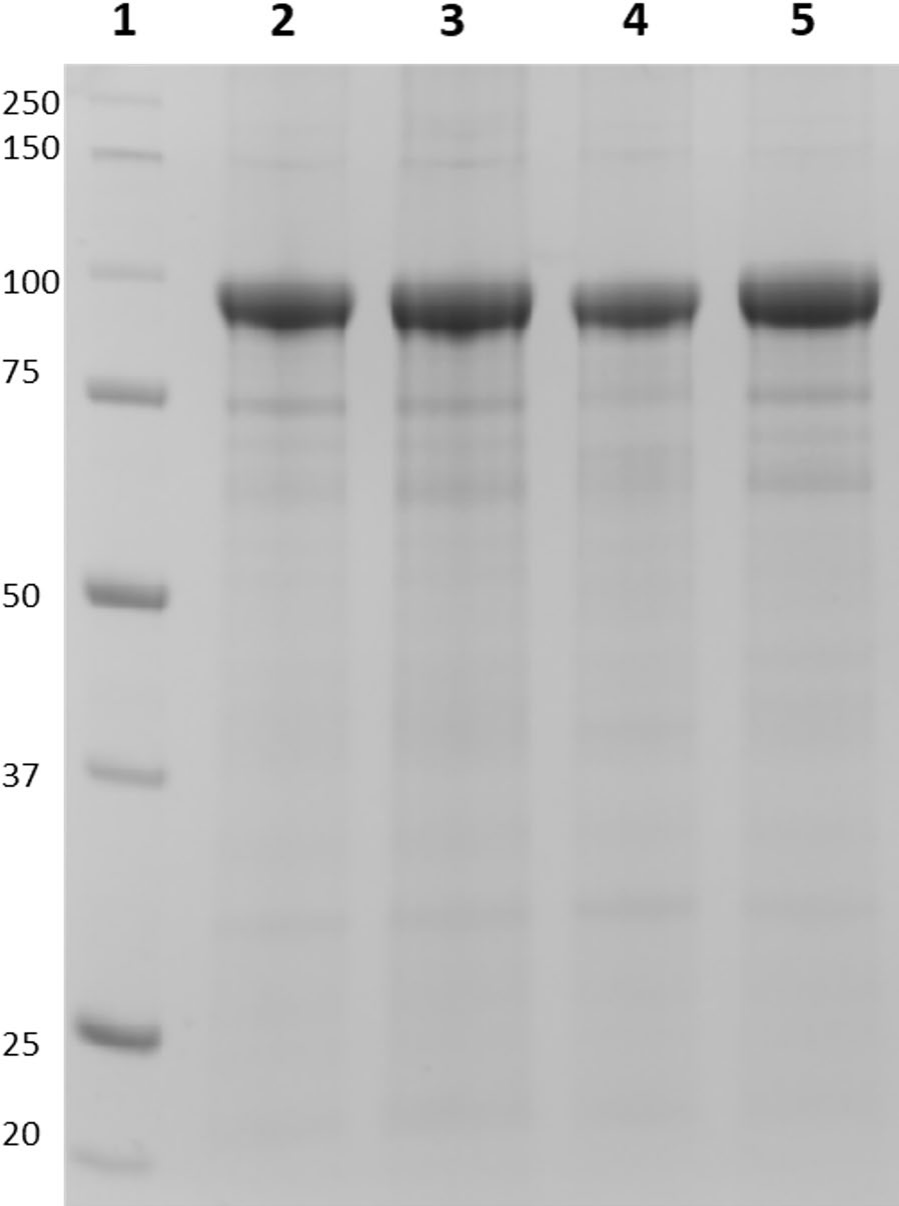
SDS-PAGE analysis of purified Cry3Aa–lipase fusion crystals. Lane (1) Molecular weight marker. Lane (2) Purified Cry3Aa–PML crystals. Lane (3) Purified Cry3Aa–DLZM4 crystals. Lane (4) Purified Cry3Aa*–PML crystals. Lane (5) Purified Cry3Aa–PML^VG^ crystals. 5-μg crystals were solubilized in 5× SDS dye, boiled and loaded onto a 10% TGX Stain-Free gel (BioRad)

Since the Cry3Aa N-terminus is known to undergo partial processing [38], the production of Cry3Aa–PML and Cry3Aa–DLZM4 was achieved by creating genetic fusions that link these lipases to the C-terminus of Cry3Aa. PML was also fused to a C-terminally truncated variant of Cry3Aa (Cry3Aa*) which was previously shown to lead to higher activity when fused to lipA [34]. Plasmids containing Cry3Aa–PML, Cry3Aa*–PML and Cry3Aa–DLZM4 were transformed and expressed in *Bt* as described previously [35]. After isolation of the crystals by density gradient centrifugation, pure Cry3Aa-fusion crystals were obtained based on SDS-PAGE analysis (Fig. 3). Unfortunately, hydrolysis of *p*-nitrophenyl palmitate (pNPP) by the fusion crystals was lower than that performed by the soluble enzymes (Table 1). Cry3Aa–PML was ~ 20-fold less active than soluble PML, and fusion to Cry3Aa* did not improve the activity. Cry3Aa–DLZM4 fared worse, displaying a nearly 700-fold lower activity than soluble DLZM4. This suggests that the mutations that stabilize soluble DLZM4 in MeOH significantly reduce its activity when immobilized as a Cry3Aa fusion. These findings support the notion that the properties obtained from directed evolution of an enzyme in solution might not necessarily translate to its immobilized state.

**Table 1:** Kinetics of soluble and immobilized lipases

Construct	Activity (U/mg)	*k*_cat_ (s^−1^)	*K*_M_ (mM)	*k*_cat_/*K*_M_ (mM^−1^ s^−1^)
Soluble PML	63.9 ± 2.5	112 ± 12	1.77 ± 0.33	63.2 ± 0.21
Soluble PML^VG^	75.5 ± 3.8	59.2 ± 2.9	0.234 ± 0.051	253 ± 0.22
Soluble DLZM4	106 ± 2.4	nd	nd	nd
Cry3Aa–PML	3.20 ± 0.34	5.89 ± 0.83	3.46 ± 0.71	1.70 ± 0.24
Cry3Aa–PML^VG^	5.86 ± 0.24	3.48 ± 0.11	0.481 ± 0.048	7.24 ± 0.10
Cry3Aa–DLZM4	0.153 ± 0.028	nd	nd	nd
Cry3Aa*–PML	1.51 ± 0.066	nd	nd	nd

One unit is defined as the amount of enzyme required to produce 1-μmol pNP per min at 25 °C. Measurements were performed in at least triplicate, and the averages are shown with the standard deviation of the mean

*nd* not determined

### Directed evolution of immobilized Cry3Aa–PML

The low activity of the immobilized Cry3Aa–PML lipases motivated us to employ a directed evolution approach to improve the immobilized catalyst activity. In light of the results for the immobilized Cry3Aa–DLZM4 mentioned above, direct screening of PML in its immobilized state was explored since any mutations that were deleterious to the immobilized enzyme’s activity, such as those in Cry3Aa–DLZM4, would be screened out immediately. As mentioned previously, one of the unique features of the CFC platform is that it is genetically encoded with the catalyst produced in its immobilized state directly in the cell. Thus, after incorporating random mutations into the PML gene, variants could be screened in vivo in their Cry3Aa-immobilized forms.

A previously developed colony screening assay [21] was used to screen immobilized Cry3Aa–PML mutants in *E. coli* for improved activity and/or stability. Although the production of Cry3Aa-fusion crystals is better in *Bt* (e.g., ease of isolation, higher yields and better purity), initial screening in *E. coli* is preferred for evolution purposes since it has higher transformation efficiency, faster growth, and does not require an intermediate strain for DNA demethylation [39]. Moreover, we have previously demonstrated that Cry3Aa*–lipA expressed in *E. coli* forms insoluble [34], but enzymatically functional particles. After a single round of mutagenesis and screening, we obtained a double mutant Cry3Aa–PML I118V, E130G (Cry3Aa–PML^VG^, Fig. 3) with improved pNPP hydrolysis activity compared to Cry3Aa–PML, resulting in a catalyst with 9% activity retention compared to soluble PML (Table 1). An additional round of directed evolution was performed and > 10,000 mutants were screened, but no mutant with higher activity was identified.

### Kinetic analyses

Kinetic analyses were performed to identify how the I118V and E130G mutations enhance the activity of PML (Table 1 and Additional file 1: Figure S1). Comparison of the two immobilized forms shows that Cry3Aa–PML^VG^ exhibits a 7.2-fold lower *K*_M_ and a 1.7-fold lower *k*_cat_ compared to Cry3Aa–PML. The soluble enzymes showed a similar trend, with the PML^VG^ mutant having a 7.6-fold lower *K*_M_ and 1.9-fold lower *k*_cat_. These data demonstrate that the improved catalytic efficiency (*k*_cat_/*K*_M_) of the PML^VG^ mutant might be due to a higher substrate-binding affinity, and that the relative improvement is not affected by fusion to Cry3Aa.

To understand the low activity of the Cry3Aa fusions, their kinetic data were compared to their soluble forms (Table 1). The *K*_M_ values of both immobilized Cry3Aa–PML and Cry3Aa–PML^VG^ were only ~ twofold higher than their soluble counterparts, suggesting that the lower activity is not due to substrate diffusion—substrate diffusional barriers are known to substantially increase the *K*_M_ of enzymes [40, 41]. In contrast, the *k*_cat_ values for Cry3Aa–PML^VG^ and Cry3Aa–PML were 17-fold and 19-fold lower than their soluble enzyme counterparts, respectively (Table 1). The similarly large decreases in *k*_cat_ suggest that the loss in the activity from both immobilized species is due to a common factor that hinders catalytic turnover. While the exact mechanism is unclear, one plausible reason is that fusion to Cry3Aa does not orient PML in an optimal position for catalysis. Notably, the activity of our previous catalyst Cry3Aa–lipA was unaffected by fusion [34]; so the activity retention of the fusion partner seems to be protein dependent. Nevertheless, we are optimistic that an alternate strategy to overcome the decrease in activity associated with fusion of PML^VG^ to Cry3Aa can ultimately be identified.

### Structure of PML^VG^ mutant

**Fig. 4:**
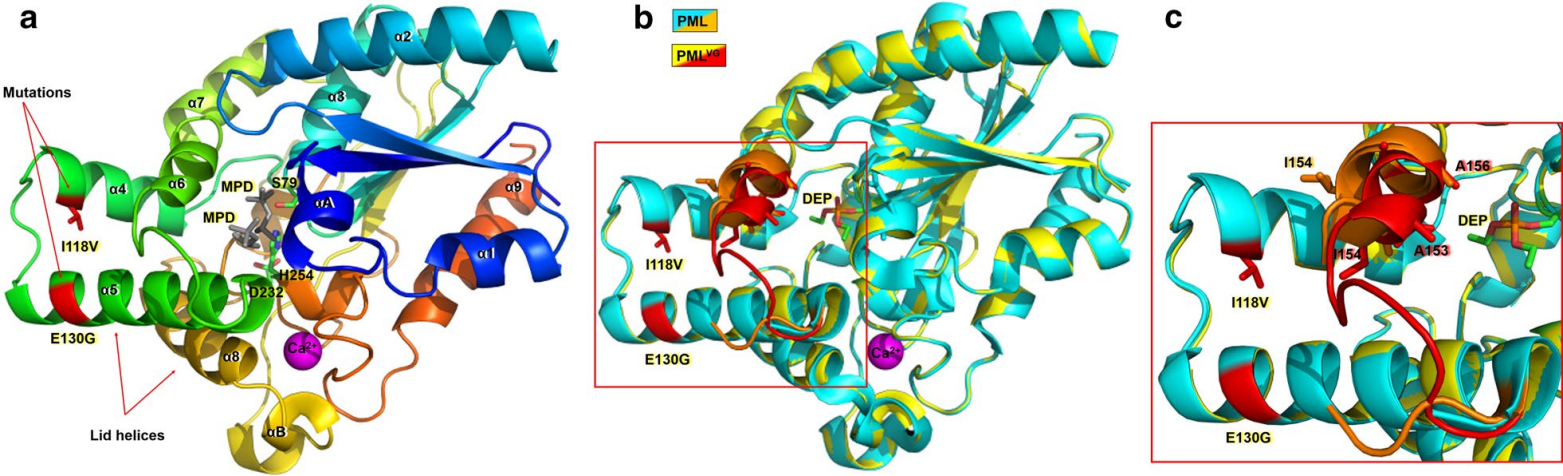
Crystal structure of PML^VG^. **a** Overall structure of PML^VG^ colored by sequence ranging from the N-terminus (blue) to the C-terminus (orange). α-Helices are labeled according to wild-type PML [43]. Lid region mutations from directed evolution are shown as red sticks and indicated with arrows. The active-site catalytic triad amino acids are labeled and shown in sticks with the carbons colored in green and the remaining atoms colored by element. A single calcium ion is colored as a magenta sphere. The two MPD molecules in the active site are shown in sticks and colored gray. **b** Alignment of PML^VG^ (yellow) to inhibitor-bound PML (PDBID: 4GXN) [43] (cyan). The DEP inhibitor is labeled and shown as sticks with the carbons colored in green and the remaining atoms colored by element. The major difference in the structure of the α6 helix between PML (orange) and PML^VG^ (red) is highlighted in the red box. **c** Close-up view showing the shift of PML^VG^ α6 hydrophobic patch toward the active site and the loop region connecting helices α5 and α6

Structural studies of the Cry3Aa–PML^VG^ crystals were attempted, but no Cry3Aa-fusion crystals could be obtained in vitro. Nevertheless, crystals of the free PML^VG^ protein were obtained and its structure was determined by X-ray crystallography to try to gain insight into how the I118V and E130G mutations impact the catalytic efficiency. The PML^VG^ structure is shown in Fig. 4a with the active-site catalytic triad, I118V and E130G mutations, α-helices and lid helices highlighted.

Previous studies of PML and its homologs have shown that this class of lipases exists in two conformations, an open conformation, and a closed conformation (Additional file 1: Figure S2). Comparison of the structures of PML^VG^ and a homologous (42%) lipase from *Pseudomonas aeruginosa* (PAL, 1EX9) reveals that the lid helix (α5) acts as a hinge that adopts a closed conformation in the absence of TAG substrates, and an open conformation upon interaction with TAG substrates [42]. Current PML structures have only been solved in closed conformations, even when bound covalently to a diethyl phosphonate (DEP) inhibitor. Even in the closed conformation, PML contains a large 9-Å channel that exposes the active site and appears to leave the oxyanion hole in a “catalytically competent” conformation [43]. This suggests that the closed conformation of PML should be active as long as the substrate is small enough to diffuse through the 9-Å channel.

Alignment of the structures of PML^VG^ and DEP-bound PML (PDB ID: 4GXN) [43] reveals that PML^VG^ is in the closed conformation as well (Fig. 4b). The overall structure is nearly identical to wild-type PML with the main structural difference being a change in helix α6 near the active site (Fig. 4c). Helix α6 contains an amino acid hydrophobic patch that has been proposed to be involved in binding to fatty acid substrates [43]. As a consequence of the two mutations, helix α6 changes from an α-helix in PML to a 3_10_ helix in PML^VG^ (Additional file 1: Figure S3) that orients its hydrophobic amino acids, particularly I154, into the substrate binding pocket and towards the DEP inhibitor (Fig. 4c). The net result is a constriction in the size of the substrate-binding channel. In spite of this tighter channel, it is likely that substrates could access the PML^VG^ active site in the absence of lid opening. This is supported by the presence of two MPD molecules in the PML^VG^ active site (Fig. 4a), and further corroborated by the positioning of the DEP inhibitor in the PML–DEP and PML^VG^ structural alignment (Fig. 4b). The PML^VG^ channel is large enough to accommodate a fatty acid substrate suggesting that PML^VG^ should be capable of hydrolyzing pNPP despite being in the closed conformation. Notably, assuming that the closed conformation was active, the tighter binding pocket in PML^VG^ compared to wild-type PML would be consistent with the lower *K*_M_ deduced from kinetics.

Another structural difference between the PML^VG^ mutant and wild-type PML is the loop region connecting helices α5 and α6. This loop is visible in the PML^VG^ structure but absent in wild-type PML (Fig. 4c) suggesting it is more disordered. Comparison of the structure of PML^VG^ to the open conformation of PAL suggests that the flexibility of this loop might be important for the dynamics of the lid to accommodate large triacylglycerol substrates (Additional file 1: Figure S2). However, taking into account the fact that the *k*_cat_ of PML^VG^ is only reduced 1.9-fold, one can conclude that either loop flexibility does not significantly impact lid dynamics when substrates bind to the enzyme in an open conformation, or it is irrelevant, because the closed conformation can directly catalyze pNPP hydrolysis reaction. The presence of a solvent channel near the active site and the fact that PML maintains a closed conformation when bound to DEP seem to support the latter case. However, hydrolysis of large triacylglycerol substrates would likely only occur in the open conformation.

### Stability of Cry3Aa–PML^VG^ crystals

**Fig. 5:**
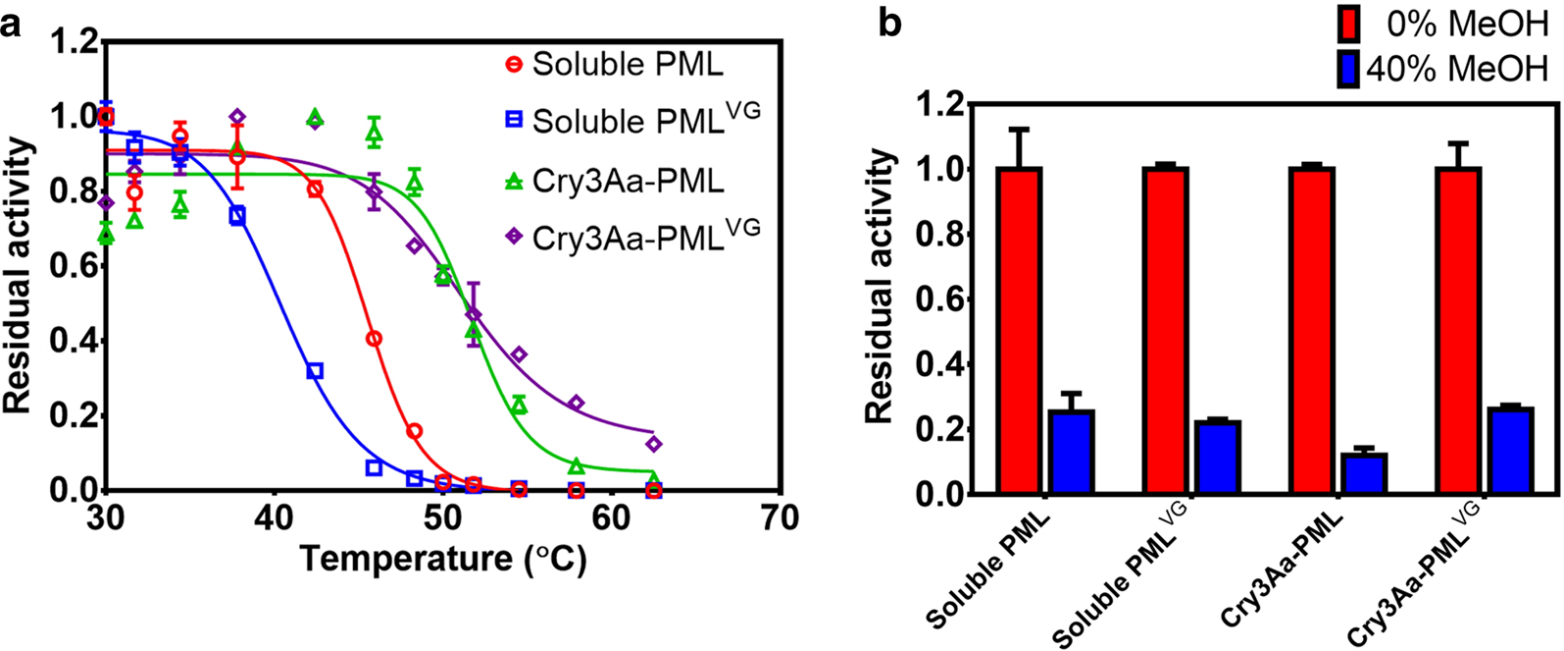
Stability of soluble and immobilized lipases. **a** Thermal stability of soluble PML, soluble PML^VG^, Cry3Aa–PML and Cry3Aa–PML^VG^. Reactions were heated for 1 h at various temperatures prior to measuring activity. Residual activity was normalized to the maximum activity for each respective construct. **b** Stability of soluble PML, soluble PML^VG^, Cry3Aa–PML and Cry3Aa–PML^VG^ in MeOH. Soluble lipases and crystals were incubated in buffer only or 40% MeOH overnight and the residual activity was normalized to the activity after overnight incubation in buffer. All reactions were performed in triplicate and error bars were derived from the standard deviation of the mean

Besides activity, another critical feature of a successful biodiesel catalyst is stability, especially against thermal and MeOH-induced inactivation. The impact of the CFC architecture on PML and PML^VG^ thermal stability was evaluated (Fig. 5a). Soluble PML and PML^VG^ had *T*_50_ values of 45.6 °C and 40.5 °C, respectively (Table 2), suggesting that the I118V and/or E130G mutations destabilize PML in solution. Cry3Aa–PML and Cry3Aa–PML^VG^ had comparable *T*_50_ values of 51.7 °C and 51.2 °C, respectively, indicating that the CFC framework stabilizes PML against thermal denaturation. Thus, the I118V and/or E130G mutations appear to destabilize PML in solution, but have no effect on PML in its immobilized state, resulting in a 10.7 °C difference in thermal stability between soluble PML^VG^ and Cry3Aa–PML^VG^. This large difference highlights the advantage of screening the enzyme in its immobilized state—the ultimate form used in FAME production. These mutations would have likely been excluded from the library of soluble PML protein variants, since they would have been screened out due to their apparent thermal instability. It is unclear why these I118V and E130G mutations destabilize PML in solution, but it is well established that incorporating flexible glycine residues internally in helices destabilizes proteins during unfolding [44]. Immobilization of PML^VG^ by Cry3Aa may counteract this by confining the configuration of PML in the Cry3Aa crystals.

**Table 2:** Stability of soluble and immobilized lipases

Construct	*T*_50_ (°C)	Residual activity after MeOH incubation (%)
Soluble PML	45.6 ± 0.38	25.3 ± 5.7
Soluble PML^VG^	40.5 ± 0.25	22.0 ± 1.1
Cry3Aa–PML	51.7 ± 0.89	12.0 ± 2.3
Cry3Aa–PML^VG^	51.2 ± 1.2	26.1 ± 1.2

Measurements were performed in at least triplicate, and the averages are shown with the standard deviation of the mean

The stabilities of soluble and Cry3Aa-immobilized lipases to MeOH were also investigated (Fig. 5b and Table 2). After incubation in MeOH, Cry3Aa–PML displayed twofold lower residual activity compared to soluble PML, indicating Cry3Aa immobilization does not confer increased stability to this polar organic solvent. This contrasts with the fact that Cry3Aa immobilization does confer thermostability to PML. On the other hand, the MeOH stability of Cry3Aa–PML^VG^, PML^VG^, and PML was very similar, indicating that, although solvent tolerance is not improved in the case of Cry3Aa–PML^VG^, the incorporation of a MeOH incubation step during screening is important to select for mutants that retain wild-type stability, and ‘weed out’ those that do not.

### Biodiesel production

With the kinetics and stability established, we proceeded to optimize the reaction parameters for FAME production from WCO by Cry3Aa–PML^VG^ crystals. The reaction conditions that can affect FAME yields include temperature, catalyst loading, MeOH:oil ratio, water content and time. Each of these parameters were optimized individually to find the best conditions for biodiesel production by Cry3Aa–PML^VG^ crystals.

**Fig. 6:**
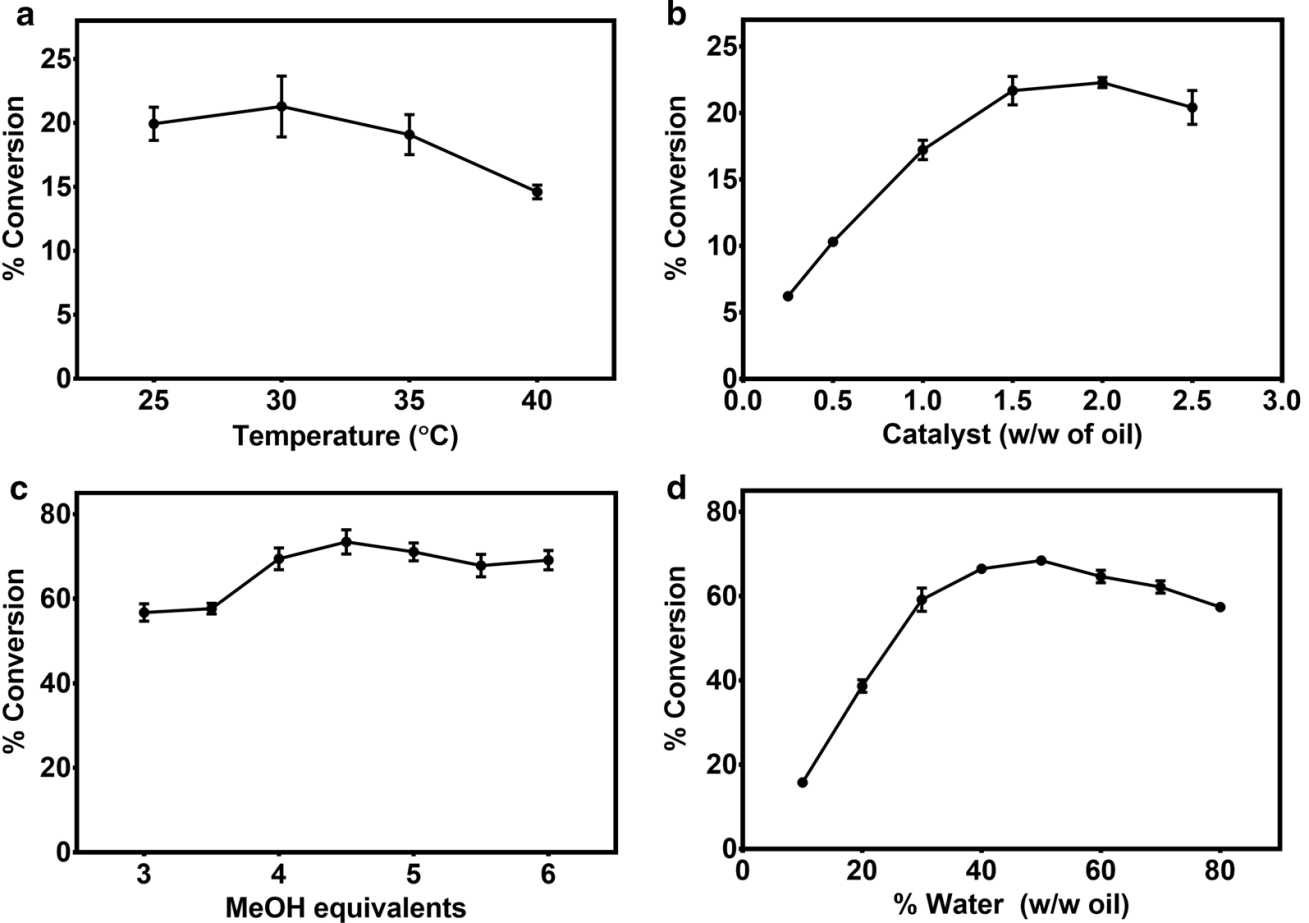
Optimization of reaction parameters during the conversion of WCO to biodiesel by Cry3Aa–PML^VG^ crystals. **a** Percent conversion as a function of incubation temperature. Reaction were incubated at 25–40 °C for 2 h. **b** Percent conversion as a function of catalyst loading. Reactions were incubated with 0.25–2.5% catalyst (w/w of oil) for 2 h. **c** Percent conversion as a function of MeOH:oil ratio. Reactions were incubated using 3–6 molar equivalents of MeOH for 24 h. **d** Percent conversion as a function of water content. Reactions were incubated using 10–80% water (w/w of oil) for 24 h. Unless otherwise indicated, all reactions contained 130-mg WCO, 1.0% w/w Cry3Aa–PML^VG^ crystals, a 5:1 MeOH:oil ratio, 40% w/w water and were incubated at 25 °C, 2000 rpm in a thermomixer. The oil layer was extracted, and the percent conversion was determined by gas chromatography (GC). All reactions were performed in triplicate and error bars were derived from the standard deviation of the mean

Previous studies have shown that PML is highly functional at low temperatures, but high temperatures resulted in poorer conversions [45]. Consistent with these findings, attempts to optimize the temperature of Cry3Aa–PML^VG^ crystals for the biodiesel reaction revealed no significant differences in FAME production from 25 to 35 °C, though the conversion dropped appreciably above 35 °C (Fig. 6a). Given that a reaction at 25 °C requires the least energy input, it was used in subsequent assays. It is uncertain why the improved thermal stability of Cry3Aa–PML^VG^ did not contribute to better conversions at higher temperatures, but it is possible that the combination of heat and MeOH is particularly deactivating to PML, or that the reduced conversions are not strictly related to enzyme stability and occur by some other temperature-induced mechanism.

Catalyst loading was varied to determine the optimum amount for WCO conversion into FAME biodiesel. As shown in Fig. 6b, biodiesel production increased almost linearly with catalyst loading, and saturated around 2% (w/w of oil) catalyst. For comparison, in many other immobilized systems, 10–30% w/w catalyst was used for biodiesel synthesis [46, 47]. The much lower loadings needed for the Cry3Aa–PML^VG^ catalyst supports the dense enzyme packing nature of CFCs.

Since MeOH equivalents can affect the efficiency of transesterification, the turnover efficiency was screened at different MeOH concentrations. At least three molar equivalents of MeOH are required to reach completion, but excess MeOH can drive the reaction forward by favoring transesterification over hydrolysis [48]. On the other hand, high MeOH concentrations can also denature enzymes; so fine-tuning is needed to identify the optimal balance. Figure 6c shows that Cry3Aa–PML^VG^ biodiesel conversion increased as the MeOH increased to 4.5 molar equivalents, but then decreased slightly at higher concentrations.

Another parameter that should be optimized is water content. Water is required to maintain enzyme structure and flexibility, but also competes with MeOH causing hydrolysis. In the case of Cry3Aa–PML^VG^, high concentrations of water had minimal effect on biodiesel conversion (Fig. 6d), which allowed for a one-step addition of MeOH since the overall MeOH concentration remained low. This is preferred to a step-wise addition of MeOH [49], since step-wise addition increases the processing steps and leads to higher costs. Concentrations of water below 30% (w/w of oil) drastically decreased the conversion, perhaps due to the poor MeOH tolerance of PML^VG^. An optimal conversion was determined to occur in 50% w/w water.

**Fig. 7:**
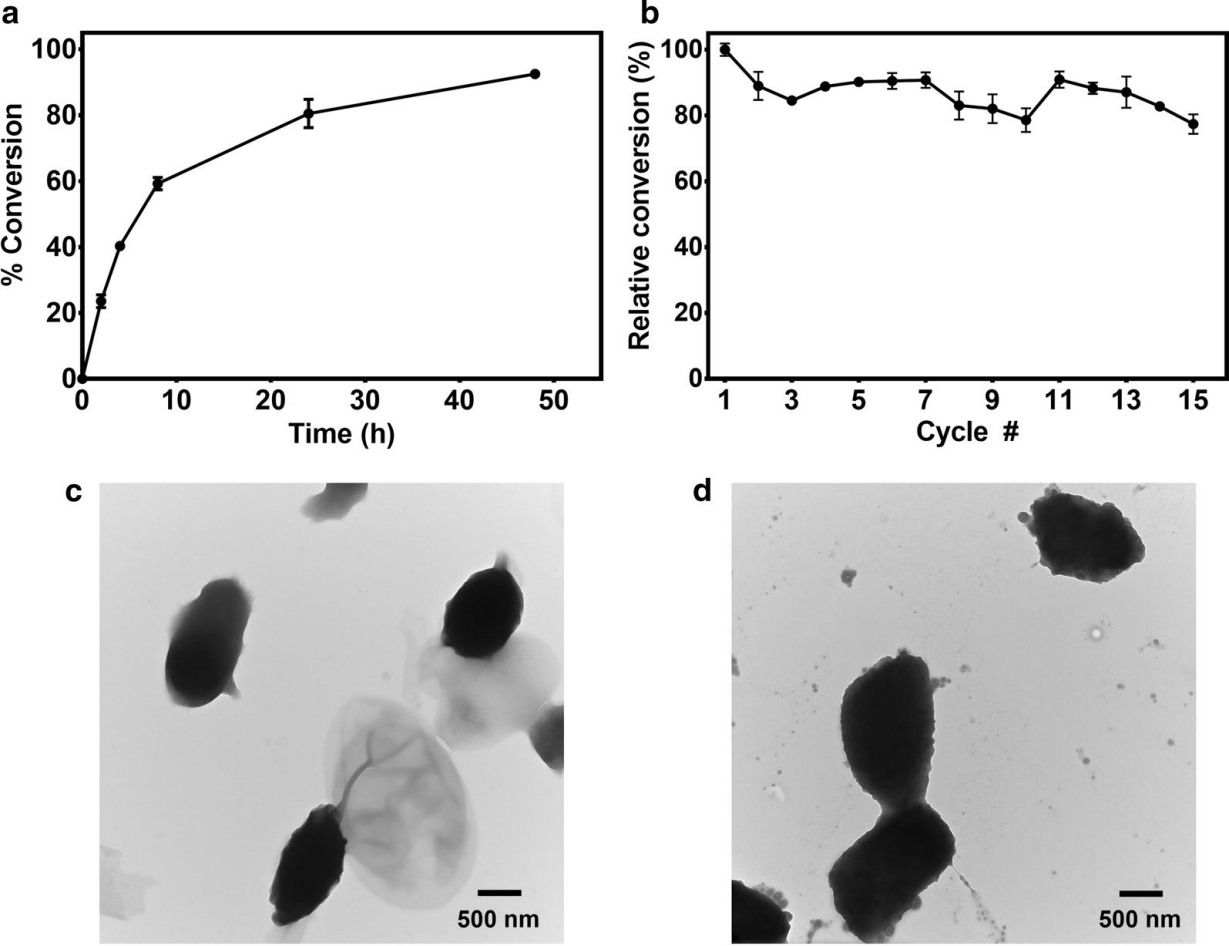
Time-point study and stability of Cry3Aa–PML^VG^ crystals during the synthesis of biodiesel from WCO. **a** A time-point study of the transesterification reaction of WCO and MeOH by Cry3Aa–PML^VG^ crystals. At 2, 4, 8, 24, and 48 h, samples were taken, and the oil layer was analyzed by GC. **b** Recyclability of Cry3Aa–PML^VG^ during the production of biodiesel from WCO using 2.0% w/w catalyst. After each cycle, the oil layer was extracted and analyzed by GC, and the crystals were washed with hexane and buffer prior to initiating the next cycle. **c** Transmission electron microscopy of Cry3Aa–PML^VG^ crystals before **d** and after 10 cycles of WCO conversion to biodiesel. Images were taken at ×25,000 and ×30,000 magnification, respectively. All reactions were performed in triplicate and error bars were derived from the standard deviation of the mean

A time-point study of Cry3Aa–PML^VG^ conversion of WCO into biodiesel was performed using the optimized parameters. After 48 h, 93% conversion could be achieved using 2% catalyst (w/w oil), 4.5 molar equivalents of MeOH, 50% water and 25 °C (Fig. 7a). This yield compares favorably to some other studies that have used WCO as a feedstock source. Yu et al. reached 79% conversion after 72 h using 40% w/w catalyst, which is 16-fold higher than the amount of catalyst used in our study [50], while Gihaz et al. used slightly more of their optimized catalyst (2.5%), and achieved a comparable yield of 80% in 24 h [51]. Thus, though we find that Cry3Aa–PML^VG^ only retains 4–5% transesterification activity of WCO relative to soluble PML^VG^ by mole, our catalyst competes well with other immobilized biodiesel catalysts for converting WCO to biodiesel in practical application. Based on our analysis, it appears that significant improvement to the performance of our catalyst can be achieved by identifying a strategy to minimize the loss of PML^VG^ activity associated with Cry3Aa fusion.

### Comparison of Cry3Aa and classical immobilization strategies

To compare the performance of the Cry3Aa-fusion approach to a classical immobilization approach, purified PML^VG^ protein was covalently conjugated onto oxirane functionalized beads (Immobead–PML^VG^) and tested for its initial rate of conversion of WCO into biodiesel. When the same amount of catalyst (1% w/w of oil) was used in the reaction, the immobilized Cry3Aa–PML^VG^ crystals exhibited a twofold higher conversion than Immobead–PML^VG^ under the same reaction conditions (Additional file 1: Figure S4). Thus, although fusion of PML^VG^ lowers its activity, the Cry3Aa–PML^VG^ catalyst has higher activity per gram than Immobead–PML^VG^ due to the dense enzyme packing nature of the Cry3Aa-fusion crystals.

### Recyclability

An important feature of any immobilized enzyme is its recyclability. After 15 cycles of use in 24-h biodiesel reactions, Cry3Aa–PML^VG^ still retained more than 77% of its initial conversion efficiency (Fig. 7b). In comparison, a recent report describing the production of biodiesel from WCO using immobilized lipase particles found that the conversion dropped to below 70% of its initial value after only 4 cycles [52]. One concern of using proteins as immobilization scaffolds is their poor mechanical stability [53, 54], but transmission electron microscopy revealed that the CFC particles did not degrade and still retained their shape after multiple rounds of conversion (Fig. 7c, d). Likely, the more resilient nature of crystals improves the catalyst’s resistance to unfolding and denaturation under harsh conditions.

## Conclusions

Directed evolution was employed to produce an immobilized lipase with improved catalytic properties. Our genetically encoded approach allowed for the first time the direct screening of the immobilized enzyme particle rather than the soluble enzyme. Since the characteristics of the soluble and immobilized enzyme can differ, direct screening of the immobilized enzyme is advantageous as evidenced by the fact that the PML mutant obtained was less stable in solution but exhibited improved stability when fused to Cry3Aa. From these evolution studies, we obtained an immobilized Cry3Aa–PML^VG^ catalyst with higher catalytic efficiency and enhanced thermal stability. Moreover, this catalyst demonstrated high conversion of WCO into biodiesel and maintained high stability and conversion over 15 reaction cycles. Not only does this work contribute to the economical production of a biodiesel catalyst for conversion of waste materials, but also it opens up a new avenue for engineering other immobilized enzymes for industrial applications.

## Methods

### Materials

The PML gene was derived from *Proteus mirabilis* (ATCC 7002) genomic DNA. The DLZM4 gene [21] was codon optimized for *Bacillus subtilis* expression and synthesized by Thermo-Scientific. WCO was collected from a local university canteen. WCO was centrifuged and filtered prior to use. MeOH, dimethylformamide (DMF) and acetonitrile (MeCN) were purchased from Millipore. Triton X-100 was purchased from Scharlau Chemicals. 1-naphthyl palmitate and Fast Blue B dye are from Santa Cruz Biotechnology. All other chemicals and materials are outlined below in parentheses.

### Construction of enzyme expression plasmids

To construct the soluble PML and soluble DLZM expression plasmids, the respective genes were amplified by Kappa HiFi DNA polymerase (Kappa Biosystems) following the manufacturer’s instructions and cloned into the pET28b vector directly downstream of the gene encoding an N-terminal His-tag using the *Nde*I and *Xho*I restriction sites via Gibson Assembly (NEB). The soluble PML^VG^ expression plasmid was constructed by amplifying PML^VG^ from the Cry3Aa–PML^VG^ plasmid (see below) and cloning it in the pET28b vector as described above. Cry3Aa–PML, Cry3Aa*–PML and Cry3Aa–DLZM4 constructs were produced by cloning the respective genes downstream of Cry3Aa or Cry3Aa* [34] in the pHT315 vector [35] using the *Bam*HI and *Kpn*I sites via Gibson Assembly. All DNA manipulations were done using XL-10 gold-competent cells, and DNA purification was achieved using the Takara MiniBEST plasmid purification kit version 4.0. Positive clones were verified by sequencing (BGI).

### Directed evolution of PML by error-prone PCR

Primers used for error-prone PCR amplification of PML contained *Nhe*I and *Hin*dIII sites at the 5′ end and 3′ end of PML, respectively, so that they could be used for T4 ligation. Error-prone PCR of PML was done using the GeneMorph II Random Mutagenesis Kit (Agilent) according to the manufacturer’s instructions for 1.5 amino acid changes per amplicon. The purified PML mutant library was digested with *Nhe*I and *Hin*dIII (NEB) and ligated downstream of Cry3Aa or Cry3Aa* in the pET28b plasmid using T4 ligation (Kapa Biosystems). After ligation, the mutant library was transformed into BL21(DE3) cells and spread on LB agar plates containing 50 µg/mL kanamycin (GoldBio).

### Screening of PML mutant library for improved activity

The screening assay was performed by following the colony lift screening protocol described previously [21]. The colonies were transferred onto filter paper (Whatman) and placed onto a LB agar plate containing 1-mM isopropyl β-d-1-thiogalactopyranoside (IPTG, IBI Scientific) and incubated at 25 °C for 2–3 h for protein expression. The cells were then lysed by immersing the filter paper in lysis buffer (50-mM NaH_2_PO_4_ pH 7.5, 0.1-M NaCl, 0.1% Triton X-100 and 1 mg/mL lysozyme) for 1 h at 25 °C. To ensure that the beneficial mutations leading to improved activity did not concomitantly perturb enzyme stability, colonies were incubated in 50% MeOH solution containing 0.1% Triton X-100 at 45 °C for 30 min. After the MeOH and heat treatment, the solution was discarded and then melted 0.5% agar containing 1-mM 1-naphthyl palmitate, 2-mM Fast Blue B, 0.5% Triton X-100, and 17% DMF was poured onto the filter paper. Colonies which formed a dark precipitate significantly faster than the control colonies were isolated from the master plate.

### Validation and combination of beneficial mutations

The BL21(DE3) colonies carrying the positive hit mutant plasmids were grown in 20 mL of LB (IBI Scientific) containing 50-µg/mL kanamycin until the OD_600_ reached 0.4–0.6. IPTG was added into the cultures at a final concentration of 0.5-mM to induce protein expression overnight at 18 °C. The cell pellets were resuspended in 1.2-mL of buffer (20-mM Tris–HCl, 100-mM NaCl, pH 7.5), and subsequently lysed by sonication. After centrifugation, the soluble and insoluble fractions were assayed for hydrolysis activity using 1-mM pNPP. Positive hits were sequenced and two PML mutants carrying I118V and E130G mutations, respectively, were cloned downstream of Cry3Aa in the pHT315 vector using the *Bam*HI and *Kpn*I sites for *Bt* expression. After verifying that both mutants had improved activity, mutations were combined using site-directed mutagenesis using Kappa HiFi to produce the pHT315 Cry3Aa–PML^VG^ plasmid.

### Expression and purification of Cry3Aa fusions and soluble enzymes

All Cry3Aa fusions were transformed, expressed and purified as previously described [35]. SDS-PAGE samples were prepared by solubilizing 30-µg crystals in 5× SDS dye and boiling for 10 min.

Soluble PML, PML^VG^ and DLZM4 proteins were expressed in *E. coli* BL21 (DE3). Transformed colonies were inoculated into 500-mL LB supplemented with 50-µg/mL kanamycin. At OD_600_ 0.8, cells were induced with 0.5-mM IPTG and incubated overnight at 16 °C, 220 rpm. Cells were harvested and resuspended in 50-mM Tris–HCl, 100-mM NaCl, pH 8.0 buffer supplemented with 1-mM phenylmethylsulfonylfluoride (Cayman Chemical) and 1-mM benzamidine hydrochloride (TCI Chemicals). All cell pellets were lysed by sonication and the cell lysate was loaded onto nickel affinity resin (GE) and eluted using an imidazole gradient (0–1 M) to yield the target proteins. Fractions containing lipase were loaded onto a SEC70 gel filtration column (BioRad) and pure elutions were combined, concentrated in a 10-kDa cut-off ultrafiltration unit (Satorius) and stored at − 80 °C.

Protein concentrations were determined using the Bradford standard assay. The Bradford reagent (BioRad) can completely dissolve the CFCs to obtain soluble protein.

### Enzyme kinetics

The specific activities were measured using pNPP (Sigma) as a substrate. A stock solution of 40-mM pNPP was prepared in a 1:1 MeCN:triton X-100 mixture. 50-µL of 2-mM pNPP prepared in 0.1-M NaH_2_PO_4_ pH 7.0 was added to 50-µL of enzyme (1–200 nM) and incubated at 25 °C. The absorbance was measured continuously at 405 nm using a Tecan M1000 and the linear portion of the graph was used to calculate the activity in μmols *p*-nitrophenol (pNP) produced (*ɛ* = 18,000 M^−1^cm^−1^) per min.

Activity retention of CFCs was determined by comparing the U/mg of the soluble enzyme to the corresponding Cry3Aa fusions after accounting for the amount of enzyme within the fusion. For example, 1-mg of Cry3Aa–PML (as determined by Bradford) would be comprised of 0.30-mg PML and 0.70-mg of Cry3Aa calculated based on their respective molecular weights. 1-U is the amount of lipase required to produce 1-μmol pNP per min at 25 °C using 1.0-mM pNPP substrate.

All kinetic parameters were determined using pNPP as a substrate. Initial rates of hydrolysis of pNPP at various concentrations (0–2.25 mM) were determined in 0.1-M NaH_2_PO_4_ pH 7.0 buffer. Enzyme concentrations used were 1-nM and 10-nM for soluble lipases and Cry3Aa lipases, respectively. The constants for *K*_M_ and *k*_cat_ were obtained from the corresponding Michaelis–Menten plots using the GraphPad Prism 6 software.

All measurements were performed in at least triplicate. The error bars show the standard deviation of the mean.

### Structure determination of PML^VG^ mutant

Crystals of PML^VG^ were obtained by vapor diffusion as sitting drops using 500-μL well solution at 18 °C. Large crystals were obtained by mixing 2-μL of 10-mg/mL PML^VG^ protein and 2-μL of well solution (0.1-M HEPES pH 7.5, 70% MPD) [43]. Crystals were soaked in crystallization condition containing 15% glycerol and flash frozen in liquid N_2_ prior to data collection.

X-ray diffraction data were collected at the National Synchrotron Radiation Research Center in Taiwan at beamline TPS 05A using a CCD detector, and the structure determined using the CCPi4 package [55]. The diffraction images were processed using iMosflm [56]. Phase determination was solved by molecular replacement in PHASER [57] using wild-type PML as a search model (PDBID: 4GW3) [43]. The model was refined using multiple rounds of Refmac5 [58] and COOT [59] over a resolution range of 19.85–1.580 Å. Data statistics can be found in Additional file 1: Table S1. The structure has been deposited with PDB ID: 6JD9.

### Thermal stability

1-μM of soluble lipases and Cry3Aa lipases were incubated in 0.1-M NaH_2_PO_4_ pH 7.0 buffer at various temperatures for 1 h in a PCR (BioRad) and then cooled to RT for 10 min. Samples were diluted and then assayed for residual activity as described in the “Enzyme kinetics” section above. Residual activity was normalized to the maximum activity observed for each respective construct. Data were fit to a sigmoidal curve and the *T*_50_ values and standard deviations were calculated using the GraphPad Prism 6 software. *T*_50_ is defined as the temperature at which 50% activity remains after 1 h incubation. All measurements were performed in triplicate and the error bars show the standard deviation of the mean.

### Methanol solvent stability

1-μM of soluble lipases and Cry3Aa lipases were incubated in 0% or 40% v/v MeOH in 60-mM NaH_2_PO_4_ pH 7.0 at 25 °C, 300 rpm for 18 h. Samples were then diluted to 4% v/v MeOH and assayed for residual activity as described in “Enzyme kinetics” section. Residual activity was normalized to the activity after incubation in 0% MeOH. All measurements were performed in triplicate and the error bars show the standard deviation of the mean.

### Immobilization of PML^VG^ onto Immobead 150P beads

Immobilization of PML^VG^ onto oxirane functional beads was done according to a previously described protocol [60]. Immobead 150P (Sigma) beads were weighed and washed with MeOH and 0.1-M NaH_2_PO_4_ pH 7.0 buffer before drying by speed vac. The beads were subsequently incubated with 1% PML^VG^ (by weight of beads) in 0.1-M NaH_2_PO_4_ pH 7.0 overnight. The amount of protein in the supernatant measured by Bradford indicated > 97% of the PML^VG^ was loaded onto the beads. The beads were washed twice with 0.1-M NaH_2_PO_4_ pH 7.0 buffer and dried before using them for transesterification reactions.

### Biodiesel reactions

Temperature optimization of biodiesel production by Cry3Aa–PML^VG^ was performed using 1.0% w/w catalyst, 130-mg WCO, 52-μL of 0.1-M NaH_2_PO_4_ pH 7 buffer (40% w/w water), and 30-μL MeOH (5:1 MeOH:oil ratio). Reactions were incubated at 25–40 °C at 2000 rpm for 2 h. The reactions were centrifuged, and the oil layer was extracted and analyzed by GC.

Optimization of Cry3Aa–PML^VG^ catalyst loading on biodiesel production was evaluated using various amounts of catalyst (0.25–2.5% w/w), 130-mg WCO, 30-μL MeOH (5:1 MeOH:oil ratio), and 52-μL of 0.1-M NaH_2_PO_4_ pH 7 buffer (40% w/w water). Reactions were incubated at 25 °C, 2000 rpm for 2 h, and then the oil layer was extracted and analyzed by GC.

Evaluation of MeOH:oil ratio on biodiesel production by Cry3Aa–PML^VG^ was done using 1.0% w/w catalyst, 130–mg WCO, and 52-μL of 0.1-M NaH_2_PO_4_ pH 7 buffer (40% w/w water). The molar equivalents of MeOH in the reactions were 3, 3.5, 4, 4.5, 5, 5.5 and 6. The reactions were incubated at 2000 rpm and 25 °C for 24 h, and then the oil layer was extracted and analyzed by GC.

The effect of water content on biodiesel production by Cry3Aa–PML^VG^ was done using 1.0% w/w catalyst, 130-mg WCO, 30-μL MeOH (5:1 MeOH:oil ratio) and 10–80% w/w water (0.1-M NaH_2_PO_4_ pH 7.0). The reactions were incubated at 2000 rpm and 25 °C for 24 h, and then the oil layer was extracted and analyzed by GC.

Transesterification as a function of time was done using 2% w/w Cry3Aa–PML^VG^ crystals in a mixture containing 390-mg WCO, 81-μL MeOH (4.5:1 MeOH:oil ratio) and 195-μL of 0.1-M NaH_2_PO_4_ pH 7 buffer (50% w/w water). Reactions were incubated at 25 °C at 2000 rpm and at 2, 4, 8, 24, and 48 h, 50-μL aliquots were centrifuged, and the oil layer was collected for GC analysis.

To compare soluble PML^VG^ and Cry3Aa–PML^VG^ transesterification rates by mole, 27-µM soluble PML^VG^ and Cry3Aa–PML^VG^ crystals were mixed with 390-mg WCO, 81-μL MeOH (4.5:1 MeOH:oil ratio) and 195-μL of 0.1-M NaH_2_PO_4_ pH 7 buffer (50% w/w water). To compare Immobead–PML^VG^ and Cry3Aa–PML^VG^ transesterification rates, 3.9-mg of crystals or beads (1.0% w/w oil) were mixed with 390-mg WCO, 81-μL MeOH (4.5:1 MeOH:oil ratio) and 195-μL of 0.1-M NaH_2_PO_4_ pH 7 buffer (50% w/w water). All reactions were incubated at 25 °C and 2000 rpm, and at 2-h and 4-h aliquots were centrifuged, and the oil layer was collected for GC analysis.

For the recyclability reaction, 7.80-mg Cry3Aa–PML^VG^ crystals (2% w/w) were added to 390-mg WCO, and the mixture was sonicated to attain homogeneity. Then, 81-μL MeOH (4.5:1 MeOH:oil) and 195-μL of 0.1-M NaH_2_PO_4_ pH 7 buffer (50% w/w water) were added to the catalyst–oil mixture and incubated in a thermomixer at 25 °C, 2000 rpm for 24 h. After each cycle, the reaction mixtures were centrifuged for 5 min and the oil layer was extracted and analyzed by GC. The Cry3Aa–PML^VG^ crystals were washed twice with 1-mL hexane and collected by centrifugation and subsequently dried in a speed-vac for 10 min. The crystals were then washed once with 1-mL of 0.1-M NaH_2_PO_4_ pH 7 buffer, centrifuged and collected before being dried in a speed-vac for 5 min. Fresh WCO, MeOH and buffer were added to initiate the next cycle. After 10 cycles, the crystals were left overnight at RT prior to washing and starting cycle 11 the next morning. All measurements were performed in triplicate and the error bars show the standard deviation of the mean.

### Quantification of biodiesel

Quantification of FAME biodiesel was performed by GC equipped with a flame ionized detector by a method described previously [34], except samples were injected into a HP-5 MS column (Agilent). The percent conversion was determined by comparing to a biodiesel sample prepared from WCO using a large excess of free *Burkholderia cepacia* lipase (Sigma) as previously described [34, 61]. Thin layer chromatography [62] was used to verify that all the WCO was converted into FAME by *B. cepacia* lipase, and I_2_ vapor was used for the staining (Additional file 1: Figure S5).

### Transmission electron microscopy

After 0 and 10 cycles of Cry3Aa–PML^VG^ catalyzed WCO conversion into biodiesel, small amounts of crystals were extracted and diluted to 0.5-mg/mL in water. Ultrathin C Type-A 400 mesh, Cu grids (Ted Pella Inc.) were dipped into the crystal solution and allowed to air dry. Samples were imaged on a Hitachi H-7650 transmission electron microscope at 80-kV and a magnification of 25,000× and 30,000× for 0 and 10 cycles, respectively. Images were taken using the AMT Camera system.

## Additional file

**Additional file 1: Figure S1.** Kinetics of soluble and immobilized lipase constructs. (a) Plot of initial rate of soluble PML and (b) soluble PML^VG^ in the presence of varying amounts of *p*-nitrophenyl palmitate (pNPP) substrate. (c) Plot of initial rate of Cry3Aa–PML and (d) Cry3Aa–PML^VG^ in the presence of varying amounts of pNPP. Rates are in nmols L^−1^ s^−1^. Experiments were performed in triplicate and the error bars represent the standard deviation of the mean. **Figure S2.** The closed and open conformations of PML. (a) Structure of PML homolog *Pseudomonas aeruginosa* lipase (PAL, 1EX9, 42% sequence ID) in the open conformation containing a bound triacylglycerol (TAG) substrate molecule. (b) Structure of PML^VG^ in the closed conformation. (c) Alignment of (a) and (b) showing how helices α5, α6 and the loop connecting these helices undergo large conformational changes to open the active site to allow large triacylglycerol (TAG) substrates to enter. The TAG substrate labeled as gray sticks is octyl-phosphinic acid 1,2-bis-octylcarbamoyloxy-ethyl ester. **Figure S3.** Comparison of α6 helix structures of PML and PML^VG^. The α6 helix in PML forms an α-helix where A153 and I154 are hydrogen bonded to L157 and E158 respectively. The α6 helix in PML^VG^ forms a 3_10_ helix where A153 and I154 are hydrogen bonded to A156 and L157 respectively. This different hydrogen bonding pattern changes the structure of the helix and the orientation of the hydrophobic amino acids lining the active site. **Figure S4.** Comparison of biodiesel production from WCO by Cry3Aa–PML^VG^ and a conventional immobilization approach. PML^VG^ was immobilized onto functional oxirane beads (Immobead–PML^VG^) and the transesterification of WCO was compared to Cry3Aa–PML^VG^ using 1% (w/w of oil) catalyst. The oil layer was analyzed by GC after reaction for 2 and 4 h. All reactions were performed in triplicate and error bars were derived from the standard deviation of the mean. **Figure S5.** Thin layer chromatography of FAME produced by *B. cepacia* lipase from waste cooking oil. (1) Waste cooking oil before and (2) after reaction with *B. cepacia* lipase. Fatty acid methyl esters (FAME), triacylglycerols (TAGS), free fatty acids (FFAs), diacylglycerols (DAGs) and monoacylglycerols (MAGs) are indicated with arrows. **Table S1.** Data collection and refinement statistics for PML^VG^ crystal structure.

## Data Availability

All data generated or analyzed during this study are included in this published article and its additional file.

## Abbreviations

*Bt*: *Bacillus thuringiensis*
CFC: Cry3A-fusion crystals
DEP: diethyl phosphonate
DLZM4: Dieselzyme 4
DMF: dimethylformamide
FAME: fatty acid methyl esters
FFAs: free fatty acids
GC: gas chromatography
IPTG: isopropyl β-d-1-thiogalactopyranoside
lipA: *Bacillus subtilis* lipase A
MeCN: acetonitrile
MeOH: methanol
MPD: 2-methyl-2,4-pentanediol
PAL: *Pseudomonas aeruginosa* lipase
pNP: *para*-nitrophenol
pNPP: *para*-nitrophenyl palmitate
PML: *Proteus mirabilis* lipase
PML^VG^: PML I118V, E130G
TAGs: triacylglycerols
WCO: waste cooking oil

## References

[1] Trusel LD, Das SB, Osman MB, Evans MJ, Smith BE, Fettweis X, McConnell JR, Noel BPY, van den Broeke MR (2018). Nonlinear rise in Greenland runoff in response to post-industrial Arctic warming. Nature.

[2] Nerem RS, Beckley BD, Fasullo JT, Hamlington BD, Masters D, Mitchum GT (2018). Climate-change-driven accelerated sea-level rise detected in the altimeter era. Proc Natl Acad Sci USA.

[3] Robles-Medina A, Gonzalez-Moreno PA, Esteban-Cerdan L, Molina-Grima E (2009). Biocatalysis: towards ever greener biodiesel production. Biotechnol Adv.

[4] Luque R, Lovett JC, Datta B, Clancy J, Campelo JM, Romero AA (2010). Biodiesel as feasible petrol fuel replacement: a multidisciplinary overview. Energy Environ Sci.

[5] Kuo TC, Shaw JF, Lee GC (2015). Conversion of crude *Jatropha curcas* seed oil into biodiesel using liquid recombinant *Candida rugosa* lipase isozymes. Bioresour Technol..

[6] Berchmans HJ, Hirata S (2008). Biodiesel production from crude *Jatropha curcas* L. seed oil with a high content of free fatty acids. Bioresour Technol..

[7] Lotero E, Liu YJ, Lopez DE, Suwannakarn K, Bruce DA, Goodwin JG (2005). Synthesis of biodiesel via acid catalysis. Ind Eng Chem Res.

[8] Hwang HT, Qi F, Yuan C, Zhao X, Ramkrishna D, Liu D, Varma A (2014). Lipase-catalyzed process for biodiesel production: protein engineering and lipase production. Biotechnol Bioeng.

[9] Mangas-Sanchez J, Adlercreutz P (2015). Highly efficient enzymatic biodiesel production promoted by particle-induced emulsification. Biotechnol Biofuels.

[10] Yu DH, Tian L, Ma DX, Wu H, Wang Z, Wang L, Fang XX (2010). Microwave-assisted fatty acid methyl ester production from soybean oil by Novozym 435. Green Chem.

[11] Bornscheuer UT, Huisman GW, Kazlauskas RJ, Lutz S, Moore JC, Robins K (2012). Engineering the third wave of biocatalysis. Nature.

[12] Schmid A, Dordick JS, Hauer B, Kiener A, Wubbolts M, Witholt B (2001). Industrial biocatalysis today and tomorrow. Nature.

[13] DiCosimo R, McAuliffe J, Poulose AJ, Bohlmann G (2013). Industrial use of immobilized enzymes. Chem Soc Rev.

[14] Worrall DM, Goss NH (1989). The formation of biologically active beta-galactosidase inclusion bodies in *Escherichia coli*. Aust J Biotechnol..

[15] Peters V, Rehm BH (2006). In vivo enzyme immobilization by use of engineered polyhydroxyalkanoate synthase. Appl Environ Microbiol.

[16] Nahalka J, Nidetzky B (2007). Fusion to a pull-down domain: a novel approach of producing *Trigonopsis variabilis*d-amino acid oxidase as insoluble enzyme aggregates. Biotechnol Bioeng.

[17] Ahmad S, Kamal MZ, Sankaranarayanan R, Rao NM (2008). Thermostable *Bacillus subtilis* lipases: in vitro evolution and structural insight. J Mol Biol.

[18] Zhang NY, Suen WC, Windsor W, Xiao L, Madison V, Zaks A (2003). Improving tolerance of *Candida antarctica* lipase B towards irreversible thermal inactivation through directed evolution. Protein Eng.

[19] Yu XW, Wang R, Zhang M, Xu Y, Xiao R (2012). Enhanced thermostability of a *Rhizopus chinensis* lipase by in vivo recombination in *Pichia pastoris*. Microb Cell Fact.

[20] Reetz MT, Soni P, Fernandez L, Gumulya Y, Carballeira JD (2010). Increasing the stability of an enzyme toward hostile organic solvents by directed evolution based on iterative saturation mutagenesis using the B-FIT method. Chem Commun.

[21] Korman TP, Sahachartsiri B, Charbonneau DM, Huang GL, Beauregard M, Bowie JU (2013). Dieselzymes: development of a stable and methanol tolerant lipase for biodiesel production by directed evolution. Biotechnol Biofuels.

[22] Tian KY, Tai K, Jian B, Chua W, Li Z (2017). Directed evolution of *Thermomyces lanuginosus* lipase to enhance methanol tolerance for efficient production of biodiesel from waste grease. Bioresour Technol..

[23] Dror A, Shemesh E, Dayan N, Fishman A (2014). Protein engineering by random mutagenesis and structure-guided consensus of *Geobacillus stearothermophilus* lipase T6 for enhanced stability in methanol. Appl Environ Microbiol.

[24] Reetz MT, Carballeira JD (2007). Iterative saturation mutagenesis (ISM) for rapid directed evolution of functional enzymes. Nat Protoc.

[25] Gao S, Zhu S, Huang R, Li H, Wang H, Zheng G (2018). Engineering the enantioselectivity and thermostability of a (+)-gamma-lactamase from *Microbacterium hydrocarbonoxydans* for kinetic resolution of vince lactam (2-azabicyclo[2.2.1]hept-5-en-3-one). Appl Environ Microbiol..

[26] Wu W, Xing L, Zhou B, Lin Z (2011). Active protein aggregates induced by terminally attached self-assembling peptide ELK16 in *Escherichia coli*. Microb Cell Fact.

[27] Lin Z, Zhou B, Wu W, Xing L, Zhao Q (2013). Self-assembling amphipathic alpha-helical peptides induce the formation of active protein aggregates in vivo. Faraday Discuss.

[28] Zhou B, Xing L, Wu W, Zhang XE, Lin Z (2012). Small surfactant-like peptides can drive soluble proteins into active aggregates. Microb Cell Fact.

[29] Diener M, Kopka B, Pohl M, Jaeger KE, Krauss U (2016). Fusion of a coiled-coil domain facilitates the high-level production of catalytically active enzyme inclusion bodies. Chemcatchem..

[30] Grage K, Rehm BH (2008). In vivo production of scFv-displaying biopolymer beads using a self-assembly-promoting fusion partner. Bioconjug Chem.

[31] Schnepf E, Crickmore N, Van Rie J, Lereclus D, Baum J, Feitelson J, Zeigler DR, Dean DH (1998). *Bacillus thuringiensis* and its pesticidal crystal proteins. Microbiol Mol Biol Rev.

[32] Whiteley HR, Schnepf HE (1986). The molecular-biology of parasporal crystal body formation in *Bacillus thuringiensis*. Annu Rev Microbiol.

[33] Sawaya MR, Cascio D, Gingery M, Rodriguez J, Goldschmidt L, Colletier JP, Messerschmidt MM, Boutet S, Koglin JE, Williams GJ, Brewster AS, Nass KJ, Hattne J, Botha S, Doak RB, Shoeman RL, DePonte DP, Park HW, Federici BA, Sauter NK, Schlichting I, Eisenberg DS (2014). Cry3A toxin structure obtained by injecting *Bacillus thuringiensis* cells in an XFEL beam, collecting data by serial femtosecond crystallographic methods and processing data with the CrystFEL software suite. Proc Natl Acad Sci USA.

[34] Heater BS, Lee MM, Chan MK (2018). Direct production of a genetically-encoded immobilized biodiesel catalyst. Sci Rep..

[35] Nair MS, Lee MM, Bonnegarde-Bernard A, Wallace JA, Dean DH, Ostrowski MC, Burry RW, Boyaka PN, Chan MK (2015). Cry protein crystals: a novel platform for protein delivery. PLoS ONE.

[36] Eggert T, Pencreac’h G, Douchet I, Verger R, Jaeger KE (2000). A novel extracellular esterase from *Bacillus subtilis* and its conversion to a monoacylglycerol hydrolase. Eur J Biochem.

[37] Chhetri AB, Watts KC, Islam MR (2008). Waste cooking oil as an alternate feedstock for biodiesel production. Energies..

[38] Li JD, Carroll J, Ellar DJ (1991). Crystal structure of insecticidal δ-endotoxin from *Bacillus thuringiensis* at 2.5 Å resolution. Nature..

[39] Lereclus D, Arantes O, Chaufaux J, Lecadet M (1989). Transformation and expression of a cloned delta-endotoxin gene in *Bacillus thuringiensis*. FEMS Microbiol Lett.

[40] Kumar S, Dwevedi A, Kayastha AM (2009). Immobilization of soybean (*Glycine max*) urease on alginate and chitosan beads showing improved stability: analytical applications. J Mol Catal B Enzym.

[41] Horvath C, Engasser JM (1974). External and internal diffusion in heterogeneous enzymes systems. Biotechnol Bioeng.

[42] Nardini M, Lang DA, Liebeton K, Jaeger KE, Dijkstra BM (2000). Crystal structure of *Pseudomonas aeruginosa* lipase in the open conformation—the prototype for family I.1 of bacterial lipases. J Biol Chem..

[43] Korman TP, Bowie JU (2012). Crystal structure of *Proteus mirabilis* lipase, a novel lipase from the proteus/psychrophilic subfamily of lipase family I.1. PLoS ONE.

[44] Serrano L, Neira JL, Sancho J, Fersht AR (1992). Effect of alanine versus glycine in alpha-helices on protein stability. Nature.

[45] Gao B, Su E, Lin J, Jiang Z, Ma Y, Wei D (2009). Development of recombinant *Escherichia coli* whole-cell biocatalyst expressing a novel alkaline lipase-coding gene from *Proteus* sp. for biodiesel production. J Biotechnol..

[46] Meunier SM, Legge RL (2012). Evaluation of diatomaceous earth supported lipase sol–gels as a medium for enzymatic transesterification of biodiesel. J Mol Catal B Enzym.

[47] Hsu AF, Jones KC, Foglia TA, Marmer WN (2004). Transesterification activity of lipases immobilized in a phyllosilicate sol–gel matrix. Biotechnol Lett.

[48] Dror A, Kanteev M, Kagan I, Gihaz S, Shahar A, Fishman A (2015). Structural insights into methanol-stable variants of lipase T6 from *Geobacillus stearothermophilus*. Appl Microbiol Biotechnol.

[49] Vahidi AK, Yang Y, Ngo TPN, Li Z (2015). Simple and efficient immobilization of extracellular his-tagged enzyme directly from cell culture supernatant as active and recyclable nanobiocatalyst: high-performance production of biodiesel from waste grease. ACS Catal..

[50] Yu CY, Huang LY, Kuan IC, Lee SL (2013). Optimized production of biodiesel from waste cooking oil by lipase immobilized on magnetic nanoparticles. Int J Mol Sci.

[51] Gihaz S, Weiser D, Dror A, Satorhelyi P, Jerabek-Willemsen M, Poppe L, Fishman A (2016). Creating an efficient methanol-stable biocatalyst by protein and immobilization engineering steps towards efficient biosynthesis of biodiesel. Chemsuschem.

[52] Wang XM, Qin XL, Li DM, Yang B, Wang YH (2017). One-step synthesis of high-yield biodiesel from waste cooking oils by a novel and highly methanol-tolerant immobilized lipase. Bioresour Technol..

[53] Sachin T, Asavari J, Gandhali J, Kamat P, Rutumbara H, Shashikant K (2013). Parameters in preparation and characterization of cross linked enzyme aggregates (CLEAs). RCS Adv..

[54] Cui JD, Jia SR (2015). Optimization protocols and improved strategies of cross-linked enzyme aggregates technology: current development and future challenges. Crit Rev Biotechnol.

[55] Winn MD, Ballard CC, Cowtan KD, Dodson EJ, Emsley P, Evans PR, Keegan RM, Krissinel EB, Leslie AGW, McCoy A (2011). Overview of the CCP4 suite and current developments. Acta Crystallogr D.

[56] Battye TGG, Kontogiannis L, Johnson O, Powell HR, Leslie AGW (2011). iMOSFLM: a new graphical interface for diffraction-image processing with MOSFLM. Acta Crystallogr D.

[57] Mccoy AJ, Grosse-Kunstleve RW, Adams PD, Winn MD, Storoni LC, Read RJ (2007). Phaser crystallographic software. J Appl Crystallogr.

[58] Vagin AA, Steiner RA, Lebedev AA, Potterton L, McNicholas S, Long F, Murshudov GN (2004). REFMAC5 dictionary: organization of prior chemical knowledge and guidelines for its use. Acta Crystallogr D..

[59] Emsley P, Lohkamp B, Scott WG, Cowtan K (2010). Features and development of Coot. Acta Crystallogr D..

[60] Alagoz D, Tukel SS, Yildirim D (2014). Purification, immobilization and characterization of (*R*)-hydroxynitrile lyase from *Prunus amygdalus turcomanica* seeds and their applicability for synthesis of enantiopure cyanohydrins. J Mol Catal B Enzym.

[61] Kaieda M, Samukawa T, Kondo A, Fukuda H (2001). Effect of methanol and water contents on production of biodiesel fuel from plant oil catalyzed by various lipases in a solvent-free system. J Biosci Bioeng.

[62] Escobar EC, Dernafelis RB, Pham LJ, Florece LM, Borines MG (2008). Biodiesel production from *Jatropha curcas* L. oil by transesterification with hexane as cosolvent. Philipp J Crop Sci..

