# Supplementary material for: Directed evolution of a genetically encoded immobilized lipase for the efficient production of biodiesel from waste cooking oil
Source: Biotechnol Biofuels. 2019 Jun 28;12:165. doi: 10.1186/s13068-019-1509-5 (PMC6598307; doi:10.1186/s13068-019-1509-5)
Supplement: Supplementary file 1 — Additional file 1: Figure S1. Kinetics of soluble and immobilized lipase constructs. (a) Plot of initial rate of soluble PML and (b) soluble PMLVG in the presence of varying amounts of p-nitrophenyl palmitate (pNPP) substrate. (c) Plot of initial rate of Cry3Aa–PML and (d) Cry3Aa–PMLVG in the presence of varying amounts of pNPP. Rates are in nmols L−1 s−1. Experiments were performed in triplicate and the error bars represent the standard deviation of the mean. Figure S2. The closed and open conformations of PML. (a) Structure of PML homolog Pseudomonas aeruginosa lipase (PAL, 1EX9, 42% sequence ID) in the open conformation containing a bound triacylglycerol (TAG) substrate molecule. (b) Structure of PMLVG in the closed conformation. (c) Alignment of (a) and (b) showing how helices α5, α6 and the loop connecting these helices undergo large conformational changes to open the active site to allow large triacylglycerol (TAG) substrates to enter. The TAG substrate labeled as gray sticks is octyl-phosphinic acid 1,2-bis-octylcarbamoyloxy-ethyl ester. Figure S3. Comparison of α6 helix structures of PML and PMLVG. The α6 helix in PML forms an α-helix where A153 and I154 are hydrogen bonded to L157 and E158 respectively. The α6 helix in PMLVG forms a 310 helix where A153 and I154 are hydrogen bonded to A156 and L157 respectively. This different hydrogen bonding pattern changes the structure of the helix and the orientation of the hydrophobic amino acids lining the active site. Figure S4. Comparison of biodiesel production from WCO by Cry3Aa–PMLVG and a conventional immobilization approach. PMLVG was immobilized onto functional oxirane beads (Immobead–PMLVG) and the transesterification of WCO was compared to Cry3Aa–PMLVG using 1% (w/w of oil) catalyst. The oil layer was analyzed by GC after reaction for 2 and 4 h. All reactions were performed in triplicate and error bars were derived from the standard deviation of the mean. Figure S5. Thin layer chromatography of FAME produce [file 13068_2019_1509_MOESM1_ESM.pdf]

Additional File 1

Directed evolution of a genetically-encoded immobilized lipase for the efficient production of biodiesel from waste cooking oil

Bradley S. Heater, Wai Shan Chan, Marianne M. Lee & Michael K. Chan*

School of Life Sciences & Center of Novel Biomaterials, The Chinese University of Hong Kong, Hong Kong SAR, China.

*Email: michaelkchan88@yahoo.com

Additional File 1

Additional Figures

Figure S1.

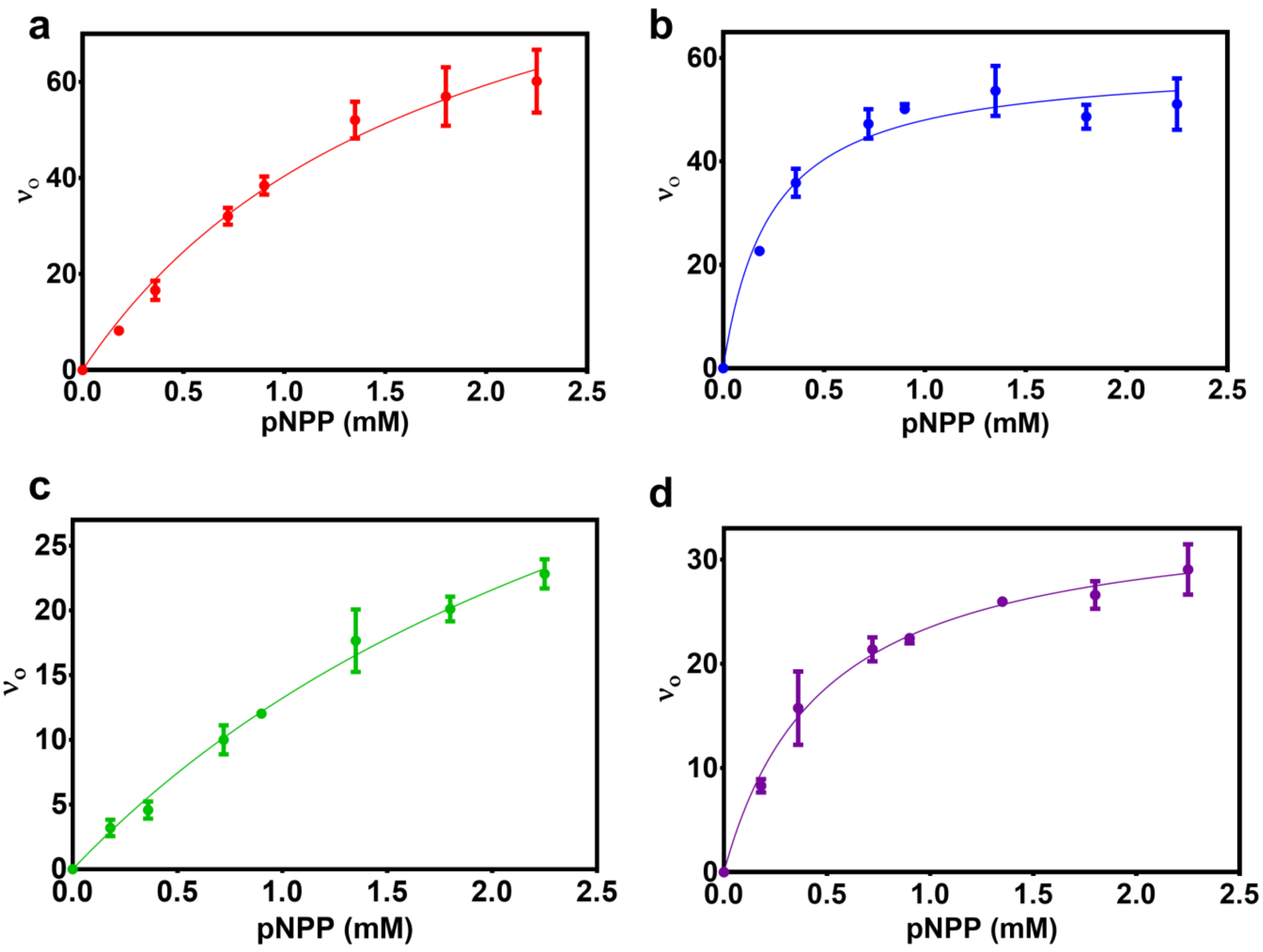

Kinetics of soluble and immobilized lipase constructs. (**a**) Plot of initial rate of soluble PML and (**b**) soluble PML^{VG} in the presence of varying amounts of *p*-nitrophenyl palmitate (pNPP) substrate. (**c**) Plot of initial rate of Cry3Aa-PML and (**d**) Cry3Aa-PML^{VG} in the presence of varying amounts of pNPP. Rates are in nmols L^{-1} s^{-1}. Experiments were performed in triplicate and the error bars represent the standard deviation of the mean.

Additional File 1

Figure S2.

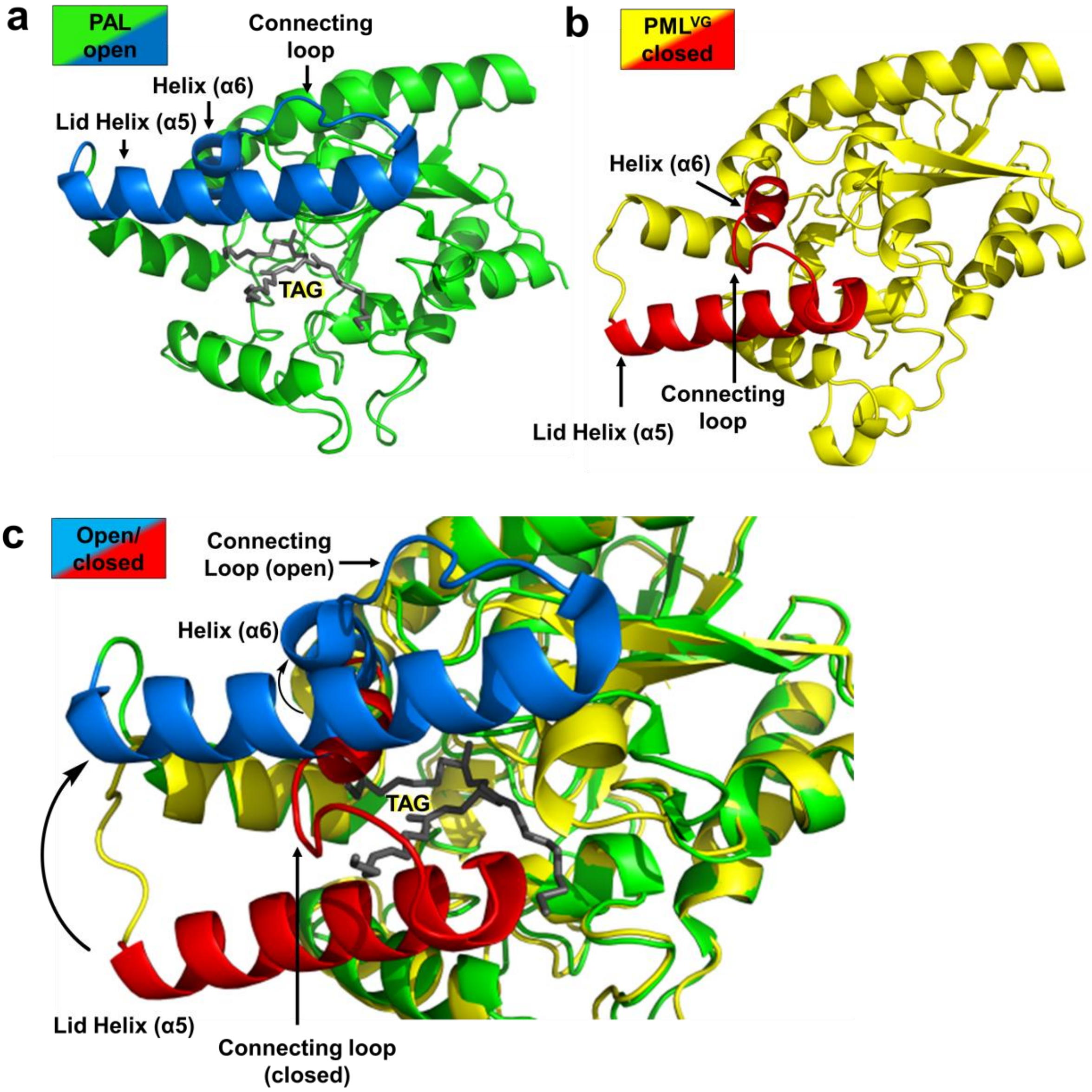

The closed and open conformations of PML. (**a**) Structure of PML homolog *Pseudomonas aeruginosa* lipase (PAL, 1EX9, 42% sequence ID) in the open conformation containing a bound triacylglycerol (TAG) substrate molecule. (**b**) Structure of PML^{VG} in the closed conformation. (**c**) Alignment of (a) and (b) showing how helices α5, α6 and the loop connecting these helices undergo large conformational changes to open the active site to allow large TAG substrates to enter. The TAG substrate labeled as gray sticks is octyl-phosphinic acid 1,2-bis-octylcarbamoyloxy-ethyl ester.

Figure S3.

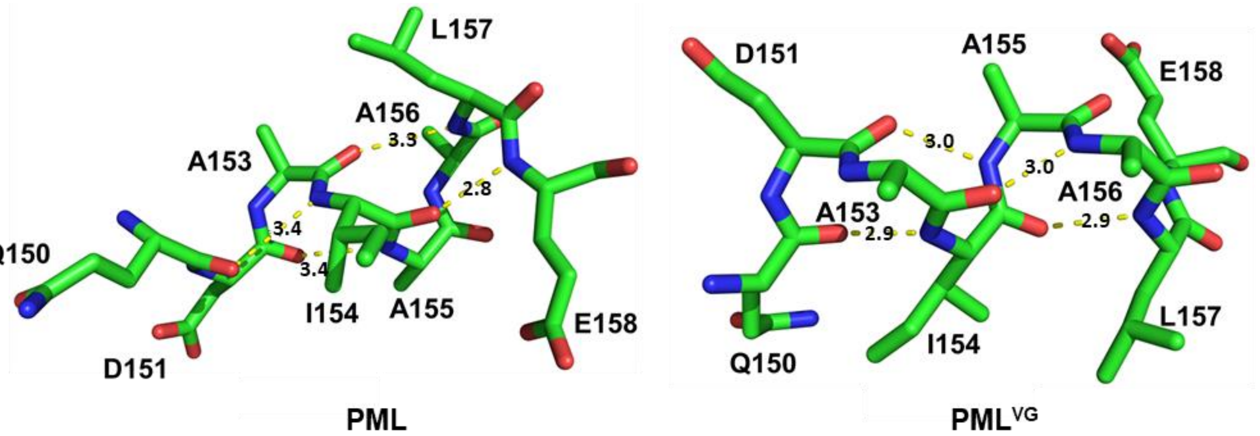

Comparison of α6 helix structures of PML and PML^{VG}. The α6 helix in PML forms an α-helix where A153 and I154 are hydrogen bonded to L157 and E158 respectively. The α6 helix in PML^{VG} forms a 3_{10} helix where A153 and I154 are hydrogen bonded to A156 and L157 respectively. This different hydrogen-bonding pattern changes the structure of the helix and the orientation of the hydrophobic amino acids lining the active site. Distances are measured in Å using PyMol.

Figure S4.

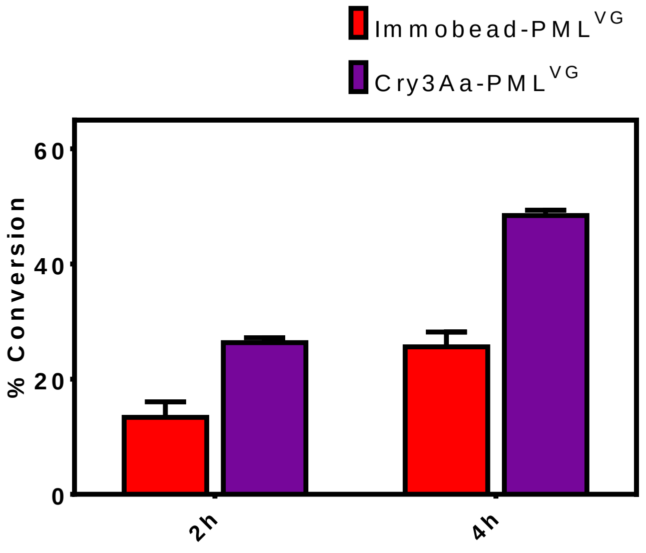

Comparison of biodiesel production from WCO by Cry3Aa-PML^{VG} and a conventional immobilization approach. PML^{VG} was immobilized onto functional oxirane beads (Immobead-PML^{VG}) and the transesterification of WCO was compared to Cry3Aa-PML^{VG} using 1% (w/w of oil) catalyst. The oil layer was analyzed by GC after reaction for 2 and 4 h. All reactions were performed in triplicate and error bars were derived from the standard deviation of the mean.

Figure S5.

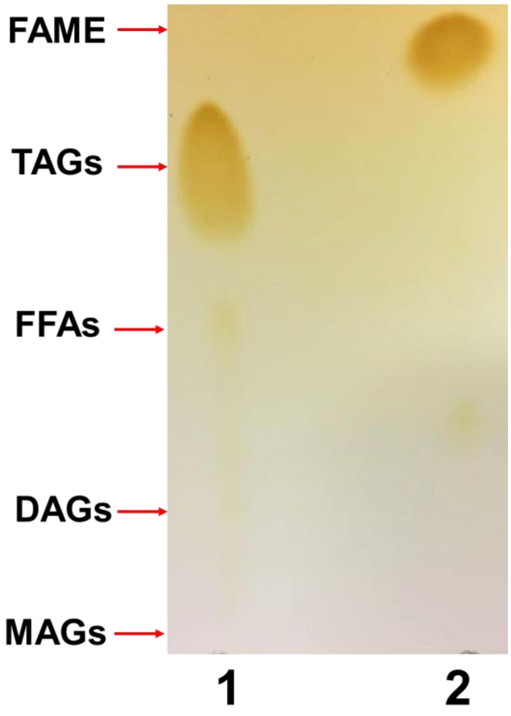

Thin layer chromatography of FAME produced by *B. cepacia* lipase from waste cooking oil (**1**) Waste cooking oil before and (**2**) after reaction with *B. cepacia* lipase. Fatty acid methyl esters (FAME), triacylglycerols (TAGS), free fatty acids (FFAs), diacylglycerols (DAGs) and monoacylglycerols (MAGs) are indicated with arrows.

Additional File 1

Table S1. Data collection and refinement statistics for PML^{VG} crystal structure.

**I. Crystallographic Data**	
Space Group	$P3_2$
Cell Dimension (Å)	a = b = 65.3, c = 63.59
Resolution (Å)	1.580
Completeness % (last shell)	100 (100)
I/σ (I) (last shell)	16.3 (10.3)
R_{merge} % (last shell)	8.2 (13.0)
**II. Refinement**	
Resolution (Å)	19.85-1.580
No. Reflections	41629
No. of atoms	2452
Average B, all atoms (Å^2)	15.0
R_{work}/Rfree %	13.9/16.3
F_oF_c correlation	0.96
**III. Geometry**	
RMS Bonds (Å)	0.0260
RMS Angles (deg°)	2.35
Ramachandran Plot Residues	
Favored (%)	278 (97.9)
Allowed (%)	5 (1.8)
Disallowed (%)	1 (0.4)
